# Hormone signaling via androgen receptor affects breast cancer and prostate cancer in a male patient: A case report

**DOI:** 10.1186/s12885-018-5216-6

**Published:** 2018-12-22

**Authors:** Haruko Takuwa, Wakako Tsuji, Masayuki Shintaku, Fumiaki Yotsumoto

**Affiliations:** Department of Breast Surgery, Shiga General Hospital, 5-4-30, Moriyama, Moriyama-City, Shiga 524-8524 Japan

**Keywords:** Androgen receptor, *BRCA* mutation, estrogen receptor, male breast cancer, prostate cancer

## Abstract

**Background:**

Male breast cancer (MBC) is rare, accounting for only around 1% of all breast cancers. Most MBCs are hormone-driven. Not only the estrogen receptor (ER), but also other steroid hormone receptors, including the androgen receptor (AR) and progesterone receptor (PgR) are expressed in MBC. AR activation in breast cancer cells facilitates downstream gene expression that drives tumorigenesis in a similar manner to ER. AR-mediated signalling works paradoxically in breast cancer and prostate cancer, and cancer cells expressing the AR are endocrine-sensitive.

**Case presentation:**

We describe a case of double cancer of MBC and prostate cancer. A 69-year-old man was referred to our hospital with a lump in his left breast in the 1990s. The patient had cT3N3M0, stage IIIC breast cancer, and underwent a mastectomy and axillary lymph node dissection. Though adjuvant chemotherapy was administered, he experienced pleural metastasis 2 months after the surgery. Two years after the recurrence during endocrine therapy with oral 5-fluorouracil, he complained of frequent urination. Radiological and histological examinations revealed that the patient had cT3N0M0, stage III primary prostate cancer with a prostate-specific antigen (PSA) level of 40.5 ng/mL. Germline mutations in the *BRCA1* and *BRCA2* genes were not tested. He received multidisciplinary, continuous therapy for both breast and prostate cancer; however, 5 and 3 years after each diagnosis, respectively, he experienced a deep vein thrombosis in his right leg related to the endocrine therapy. Liver metastasis progressed after he stopped breast cancer therapy. However, long-term disease control had been achieved with anti-estrogen therapy for breast cancer and estrogen replacement therapy for prostate cancer.

**Conclusions:**

Several studies have shown that estrogen exposure after estrogen depletion likely causes apoptosis of ER-positive breast cancer cells. Our findings indicate that this also applies to the environment in male body. AR dominant signaling prevents breast cancer recurrence and metastasis, especially in MBC patients.

## Background

Male breast cancer (MBC) accounts for only around 1% of all breast cancers [1–4]. MBC has high rates of hormone receptor expression; approximately 90–95% of all MBCs express the estrogen receptor (ER), 80–81% express the progesterone receptor (PgR), and 34–87% the androgen receptor (AR) [5–10]. Human epidermal growth factor receptor 2 (HER2)-positive MBC is rare [11].

Factors associated with an increased MBC risk are related to several genetic disorders, such as Kleinfelter’s syndrome, *BRCA 1/2*, *PTEN*, *p53*, and *CHEK2* mutations [12–17], and hormonal alterations, in particular, estrogen and androgen imbalance [15]. AR-positive patients (including female breast cancer and triple-negative breast cancer patients) respond to endocrine therapy, and have better prognoses [5, 18]. On the other hand, androgens are important for the development and maintenance of prostate cancer cells [19]. The biological function of androgens is exerted through the activation of the transcriptional activity of the AR [20–22].

Herein, we present the case of a patient with advanced MBC who experienced advanced prostate cancer during systemic therapy for metastatic MBC.

## Case report

A 69-year-old man presented to the outpatient clinic of the Department of Breast Surgery at the Shiga General Hospital (Moriyama, Shiga, Japan) with a lump in his left breast in the 1990s. Physical examination revealed a mass measuring > 6 cm without skin invasion in the upper-lateral region as well as axillary lymph node swelling. The patient had a history of diabetes, hyper tension, cerebral infarction, and brain schwannoma controlled by oral glimepiride 0.5 mg, nifedipine 40 mg, and aspirin 100 mg. He had no remarkable family history.

**Fig. 1:**
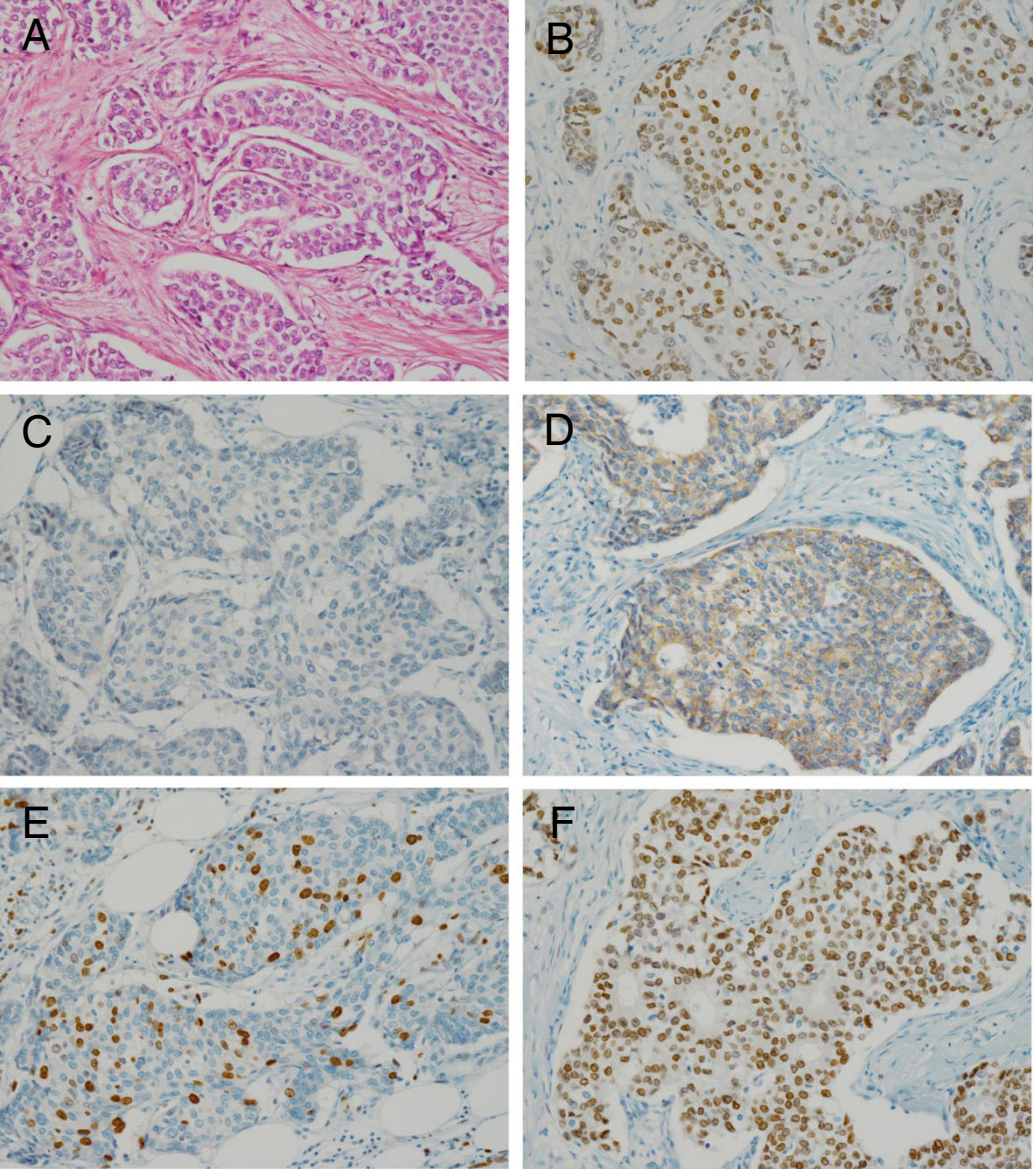
**a** Representive photomicrographs of the breast cancer (hematoxylin-eosin staining: magnification, × 200). Pathological examination defined the mass as invasive ductal carcinoma, histological grade 3; (**b**) Estrogen receptor-positive: 90%; (**c**) Progesterone receptor-negative: 0%; (**d**) Human epidermal growth factor receptor 2-negative: score 1+; (**e**) Ki-67 labeling index, 10%; (**f**) Androgen receptor-positive: 95%

Ultrasonography showed a breast mass and right-sided axillary lymph node swelling. A core needle biopsy was performed, and the tumor was diagnosed as a high histological grade invasive ductal carcinoma. It was ER-positive, PgR-negative, HER2-negative and AR-positive (detected by anti-AR rabbit monoclonal antibody SP107; Roche tissue diagnostics, Ltd.); the Ki-67 labeling index was 10% (Fig. 1). The tumor was radiologically classified as cT3N3M0, stage IIIC disease (Union for International Cancer Control-TNM classification, 8th edition) [23].

Preoperative chemotherapy was not performed at the time; thus, a mastectomy and axillary dissection were performed. The final histological diagnosis was pt3n3a (36/39) M0, stage IIIC disease. Postoperative chemotherapy with epirubicin 40 mg/body with 5-fluorouracil (5-FU) 500 mg/body every 2 weeks plus oral cyclophosphamide 100 mg daily (CEF) was administered. After 2 cycles of the chemotherapy, computed tomography (CT) revealed pleural metastasis in his right lung. Anti-estrogen therapy with high-dose toremifene (TOR) 120 mg b.i.d and oral 5-FU, doxifluridine (5’DFUR) 1200 mg was administered as first-line therapy for metastatic breast cancer.

**Fig. 2:**
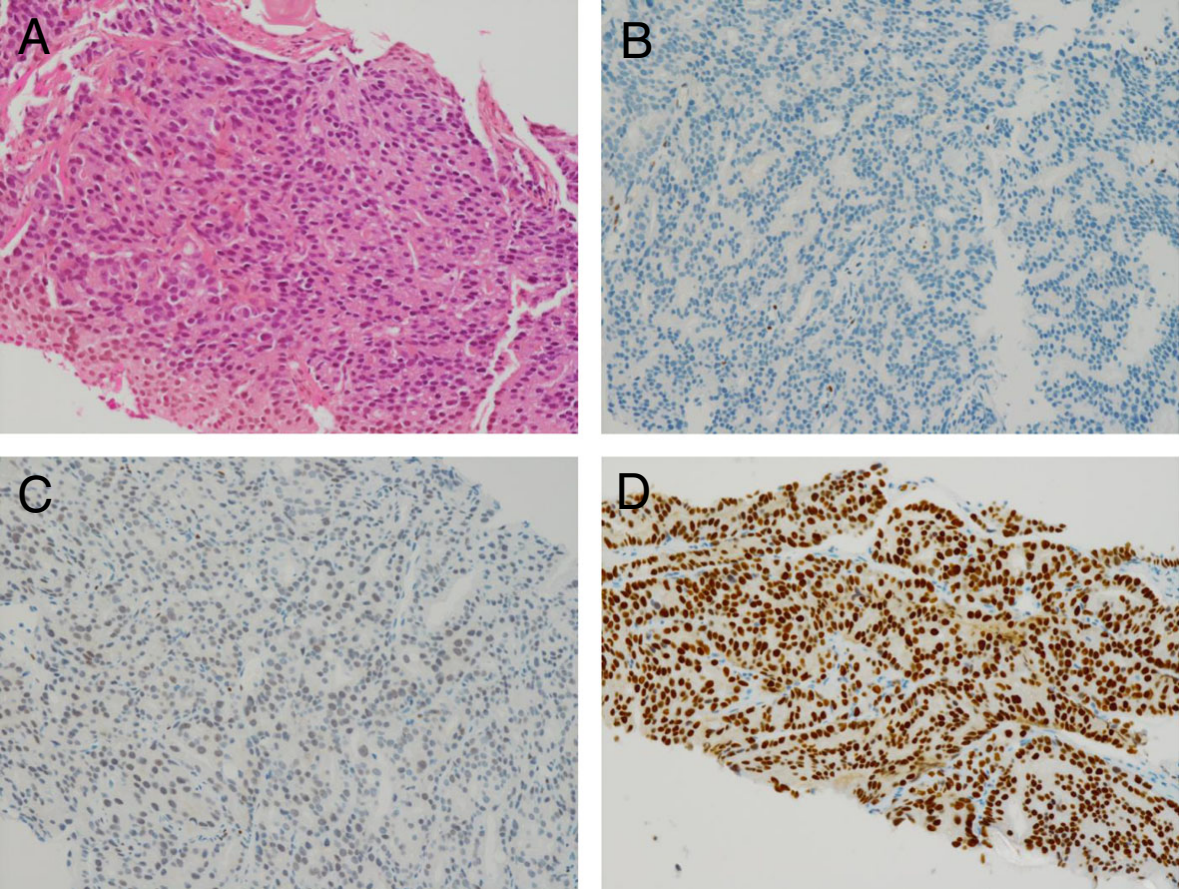
**a** Representive photomicrographs of the prostate cancer (hematoxylin-eosin staining: magnification, × 200). Glandular fusion is the hallmark feature of a Gleason score of 8. The image shows fused glands forming anastomosing irregular cords separated by small amounts of stroma. **b** Estrogen receptor-positive: 3%; (**c**) Progesterone receptor-negative: 0%; (**d**) Androgen receptor-positive: 95%

Two years after the recurrence, the patient complained of frequent urination. Radiological and histological examinations revealed that he had cT3N0M0, stage III primary prostate cancer (Fig. 2) with a prostate-specific antigen (PSA) level of 40.5 ng/mL. He underwent radiation therapy at a dose of 66.0 Gy/33 fractions as local control to treat the prostate cancer. After radiotherapy, the prostate cancer therapy was temporarily suspended, and the watchful waiting approach was taken. Breast cancer therapy with high-dose TOR was continued since breast cancer control was good. Six months later, the PSA level increased from 3.7 ng/mL to 18.0 ng/mL. Oral estramustine phosphate sodium hydrate (Estracyt®) 626.8 mg was administered daily because this drug remains specifically in prostate cancer tissue and is not contraindicated in patients with breast cancer. The PSA level immediately decreased to within the normal range (3.7 ng/mL), and Estracyt® as well as TOR were continued for 3 years until the patient experienced a deep vein thrombosis in his right leg related to the endocrine therapy, while the antiplatelet therapy was ongoing.

**Fig. 3:**
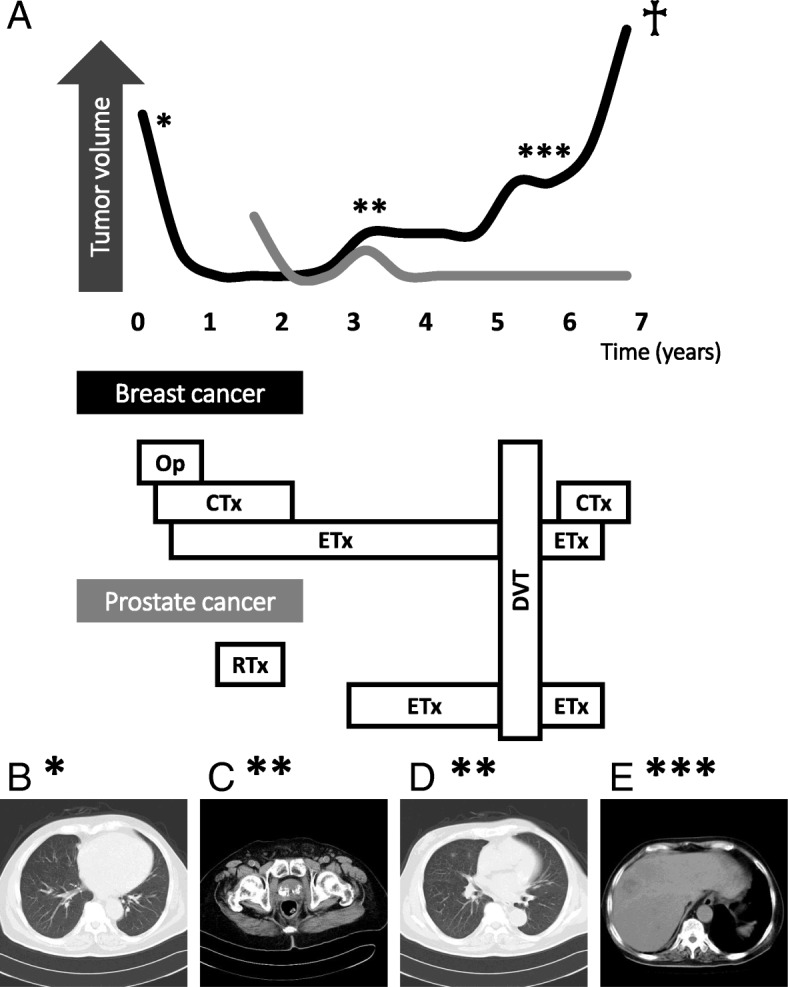
Clinical course in the patient. **a** History of breast and prostate cancer treatment; (**b**) The images show (*) when the patient was diagnosed with pleural metastasis; (**c**) (**) for prostate cancer recurrence with prostate-specific antigen elevation; and (**d**) (**) for new regions of lung metastases. **e** (***) The patient experienced liver metastasis when he temporarily stopped systemic therapy for breast cancer. Abbreviations: Op = operation; CTx = chemotherapy; ETx = endocrine therapy; RTx = radiotherapy; DVT = deep venous thrombosis

TOR was stopped but Estracyt® was continued for symptomatic disease control. A CT scan revealed liver metastasis from the breast cancer after the patient stopped the breast cancer therapy. Estracyt® was changed to the non-steroidal anti-androgen agent, Casodex® as the second-line endocrine therapy for the prostate cancer and a luteinizing hormone-releasing hormone (LHRH) agonist as third-line treatment; however, the patient died due to breast cancer progression 5 years after the diagnosis of prostate cancer (Fig. 3).

We retrospectively reviewed the medical records of 1431 patients with breast cancer who underwent breast cancer therapy at the Shiga General Hospital between 1998 and 2017. The retrospective review of the medical records was approved by the appropriate ethics review board, and the study complied with the tenets of the Declaration of Helsinki. Of all patients, 8 (0.6%) were men; they were diagnosed with primary breast cancer histologically. Table 1 shows the clinicopathological characteristics of the patients with MBC.

**Table 1:** Clinicopathological characteristics of the patients with male breast cancer

age	TNM classification	histology	ER (%)	PgR (%)	HER2 (FISH)	AR (%)	Ki-67 (%)	Histological grade	Radiation	Adjuvant therapy	Follow up (months), Outcome
47	T2N0M0	IDC	99	80	1+	60	50	2 (2/3/1)	–	pre; TC (4) post; cape+TAM	8
58	T1bN0M0	IDC	99	40	1+	20	20	2 (3/2/1)	–	post; TAM	27
63	T1aN0M0	IDC	99	80	1+	90	10	1 (2/2/1)	60Gy/30fr (WBRT)	–	48
63	T1cN0M0	IDC	90	80	2+ (1.54)	95	70	3 (3/2/3)	–	post; TAM	90
69	T3N3aM0	IDC	90	0	1+	95	10	3 (3/3/2)	–	post; CEF (2) MBC; TOR + 5’DFUR wPTX	76 deceased
72	T2N3aM0	IDC	99	90	2+ (2.26)	90	30	2 (3/2/1)	50Gy/25fr (PMRT)	pre; wPTX + trastuzumab (4) post; TAM + trastuzumab (13)	14
74	T2N0M0	IDC	95	95	0	95	15	1 (2/2/1)	–	post; TAM	56 Local recurrence, bone meta at 24 months
74	T2N0M0	mucinous	80	80	0	80	< 1	–	–	post; TAM	57

## Discussion

We report the case of a patient with both, MBC and prostate cancer, in whom using Estracyt® as estrogen replacement therapy after anti-estrogen therapy with high-dose TOR resulted in better control of breast cancer than prostate cancer for a few years.

Men with *BRCA1/2* mutations are at increased risk for breast, prostate, pancreatic and other cancers [2, 3, 14, 16]. Especially in individuals with the *BRCA2* mutation, prostate cancer is the most commonly diagnosed cancer, followed by MBC [16]. Though a *BRCA2* mutation was strongly suspected in this case based on the history of the patient (diagnosis with high-grade histology and advanced-stage breast and prostate cancer), genetic testing was not available 20 years ago. Because the patient was already deceased, a germ line mutation was not tested. Previous studies showed that MBC with a *BRCA* mutation is sensitive to systemic therapy, and patients have a favorable overall survival [14, 16, 24].

Though the dose of CEF that the patient received was lower than the doses prescribed recently, the primary breast cancer of this patient was potentially chemotherapy-resistant [25, 26]. The metastatic site of the right pleura was small and asymptomatic, and the cancer cells were endocrine therapy-naïve and were thus expected to be endocrine-sensitive and anthracycline-resistant. High-dose TOR with oral 5-FU were administered as first-line therapy for the metastatic breast cancer. At the time, the options of endocrine therapy were tamoxifen or TOR as selective estrogen receptor modulators (SERM). High-dose TOR was recommended as it was expected to be more effective for cancer that is primary therapy-resistant [27–29].

Generally, SERMs have no predictive marker that indicates their biological efficiency, unlike LHRH agonists and AI do with the estrogen level. Furthermore, the active metabolite of tamoxifen, endoxifen is generated through hepatic enzyme chytochrome P450 (CYP) CYP2D6 and CYP3A. And genetic variants of CYP2D6 may affect response to tamoxifen [30]. On the other hand, a higher preoperative serum estradiol (E2) level was reported to be a negative prognostic factor in female breast cancer patients without E2 depletion [31]. Estrogen exposure after estrogen depletion was observed to cause apoptosis of ER-positive breast cancer cells in several studies in both pre- and postmenopausal women [32–34]. Our patient was exposed high serum levels of estrogen in the form Estracyt® as prostate cancer therapy after 3 years of SERM. The serum level of E2 which, among the estrogens, has the strongest physiological activity as a stimulator in ER-positive breast cancer, ranges between 20 pg/mL and 60 pg/mL in healthy men. Surprisingly, the E2 level increases up to 2–6 ng/mL during Estracyt®treatment [35]. In our patient, the metastases were relatively controllable during treatment with high-dose TOR and Estracyt®. This finding in line with the suppression of cancer cell proliferation regardless of E2 level dynamics in premenopausal patients with breast cancer undergoing SERM therapy. After a 3-month break of systemic therapy for metastatic breast cancer, high-dose TOR was re-administered; however, it was not effective for the new liver metastasis. Importantly, first line-therapy for metastatic breast cancer had been effective for 5 years.

Another mechanism was dependent on AR-mediated downregulation of tumorigenic signal transduction. AR-positive patients, including female breast cancer and triple-negative breast cancer patients, respond to endocrine therapy and have better prognoses [5, 18]. In human prostate cancer, ER-β is silenced in cancers that are not well differentiated [36]. The AR causes the proliferation and secretion of prostate tissue whereas ER-β suppresses its proliferation and promotes differentiation. The prostate cancer did not express ER in this patient (Fig. 2b) when he underwent prostate biopsy during anti-estrogen therapy for metastatic breast cancer. The AR works paradoxically between breast cancer cells and in prostate cancer cells. The AR and PgR have similar structures, and the AR mediates tumorigenic signal downregulation in castration-resistant prostate cancer [37, 38]. Anti-androgen therapy was not performed as the initial systemic therapy for prostate cancer in this patient; it may have prolonged the control of both breast cancer and prostate cancer. After exposure to endocrine therapy, some mutation related with endocrine-resistance in cancer cells are detected by sequencing or molecular testing. The weakness of this study was that such testing was not performed for this patient. However, estrogen exposure after estrogen depletion might be effective strategy for ER-positive metastatic breast cancer in such situation with endocrine-resistance.

All patients with MBC patients expressed the ER and AR (Table 1). Besides the patient of the case report, one patient experienced local recurrence due to biopsy tract implantation. All patients had a relatively long prognosis although cases of locally advanced breast cancer and high histological grade disease were included. This might indicate a better endocrine sensitivity of AR-expressing breast cancer.

Endocrine therapy for both breast and prostate cancer is difficult to balance both diseases. This is because endocrine therapy for breast cancer and prostate cancer acts sometimes cooperatively, and sometimes against with conflicting mechanisms. Again, we would like to emphasize our case underwent estrogen exposure after estrogen depletion, and this strategy might be effective for ER- and AR-positive metastatic breast cancer patients.

## Conclusion

The patient of this report who had double, breast and prostate cancer, showed good long-term control of both diseases. He may have had a *BRCA* mutation. AR-mediated signalling works paradoxically in breast cancer and prostate cancer, and cancer cells expressing the AR are endocrine-sensitive. Thus, high-dose TOR and Estracyt® after high dose TOR were effective in this case.

## Abbreviations

5’DFUR: 5-fluorouracil, doxifluridine
5-FU: 5-fluorouracil
AR: androgen receptor
CEF: cyclophosphamide, epirubicine and 5-fluorouracil
CT: compute tomography
E2: estradiol
ER: estrogen receptor
HER2: human epidermal growth factor receptor 2
LHRH: luteinizing hormone-releasing hormone
MBC: male breast cancer
PgR: progesterone receptor
PSA: prostate-specific antigen
SERM: selective estrogen receptor-modulator
TOR: toremifene
